# Immune thrombocytopenia (ITP) World Impact Survey (iWISh): Patient and physician perceptions of diagnosis, signs and symptoms, and treatment

**DOI:** 10.1002/ajh.26045

**Published:** 2020-12-19

**Authors:** Nichola Cooper, Alexandra Kruse, Caroline Kruse, Shirley Watson, Mervyn Morgan, Drew Provan, Waleed Ghanima, Donald M. Arnold, Yoshiaki Tomiyama, Cristina Santoro, Marc Michel, Serge Laborde, Barbara Lovrencic, Ming Hou, Tom Bailey, Gavin Taylor‐Stokes, Jens Haenig, James B. Bussel

**Affiliations:** ^1^ Department of Haematology Hammersmith Hospital, Imperial College London London UK; ^2^ Platelet Disorder Support Association Cleveland Ohio USA; ^3^ Patient Representative for the UK ITP Forum Bolnhurst UK; ^4^ ITP Support Association Bolnhurst UK; ^5^ Academic Haematology Unit, Blizard Institute Barts and The School of Medicine and Dentistry London UK; ^6^ Department of Medicine Østfold Hospital Trust Kalnes Norway; ^7^ Department of Hematology Institute of Clinical Medicine, University of Oslo Oslo Norway; ^8^ Department of Medicine, McMaster Centre for Transfusion Research McMaster University Hamilton Ontario Canada; ^9^ Department of Blood Transfusion Osaka University Hospital Osaka Japan; ^10^ Hematology University Hospital Policlinico Umberto I Rome Italy; ^11^ Department of Internal Medicine, National Referral Center for Adult Immune Cytopenias Henri Mondor University Hospital, Assistance Publique Hôpitaux de Paris, Université Paris‐Est Créteil Créteil France; ^12^ O'Cyto Saint‐Loubes France; ^13^ Italian Association of Immune Thrombocytopenic Purpura Caprino Veronese Italy; ^14^ Department of Hematology Shandong University Jinan China; ^15^ Bespoke Team Adelphi Real World Macclesfield UK; ^16^ Novartis Pharma AG Basel Switzerland; ^17^ Division of Hematology/Oncology Weill Cornell Medicine New York New York USA

## Abstract

Immune thrombocytopenia (ITP) is now well‐known to reduce patients' health‐related quality of life. However, data describing which signs and symptoms patients and physicians perceive as having the greatest impact are limited, as is understanding the full effects of ITP treatments. I‐WISh (ITP World Impact Survey) was an exploratory, cross‐sectional survey designed to establish the multifaceted impact of ITP, and its treatments, on patients' lives. It focused on perceptions of 1507 patients and 472 physicians from 13 countries regarding diagnostic pathway, frequency and severity of signs and symptoms, and treatment use. Twenty‐two percent of patients experienced delayed diagnosis (caused by several factors), 73% of whom felt anxious as a result. Patients rated fatigue among the most frequent, severe symptom associated with ITP at diagnosis (58% most frequent; 73% most severe), although physicians assigned it lower priority (30%). Fatigue was one of the few symptoms persisting at survey completion (50% and 65%, respectively) and was the top symptom patients wanted resolved (46%). Participating physicians were experienced at treating ITP, thereby recognizing the need to limit corticosteroid use to newly‐diagnosed or first‐relapse patients and espoused increased use of thrombopoietin receptor agonists and anti‐CD20 after relapse in patients with persistent/chronic disease. Patient and physicians were largely aligned on diagnosis, symptoms, and treatment use. I‐WISh demonstrated that patients and physicians largely align on overall ITP symptom burden, with certain differences, for example, fatigue. Understanding the emotional and clinical toll of ITP on the patient will facilitate shared decision‐management, setting and establishment of treatment goals and disease stage‐appropriate treatment selection.

## 1 INTRODUCTION

Primary immune thrombocytopenia (ITP) is an autoimmune disorder characterized by reduced platelet counts (<100 × 10^9^/L) and increased bleeding risk in the absence of other causes associated with thrombocytopenia.^1^ ITP may affect patients' lives in many ways, including not only hemorrhagic manifestations, fear of bleeding, and secondary complications that can be associated with therapeutic options, but especially anxiety of the unknown (common in chronic diseases) and reduced energy levels.^2^ Severe bleeding is rare in ITP,^3^ but more frequent in elderly patients.^4^, ^5^ Paradoxically, patients with ITP are at risk of thromboembolic events.^4^ Important symptoms often reported by patients when asked, but which can be easily overlooked, involve the effects of ITP on health‐related quality of life (HRQoL), including unexplained fatigue (up to 45%),^4^, ^6^, ^7^, ^8^ anxiety or depression (29%), and headache (16%).^7^

Corticosteroids, intravenous immunoglobulin (IVIg), and anti‐D immunoglobulin are standard first‐line treatment options for patients with newly‐diagnosed primary ITP.^9^ None are recommended for use beyond 6‐12 weeks, because of lack of curative effects, high costs with transient benefit and drug‐related complications. Most patients with primary ITP will relapse after first‐line treatment discontinuation.^10^ An unknown proportion of adults with newly‐diagnosed ITP will improve (with or without treatment) by 1 year from diagnosis, with estimates ranging from 13% of patients to 60% being better within 3 years.^11^, ^12^

In the absence of head‐to‐head trials, rational determination of the most appropriate second‐line treatment choice for patients with persistent/chronic ITP is not possible.^2^, ^12^, ^13^ Consequently, second‐line treatment is individualized according to patient and physician preferences,^13^, ^14^ based on single‐agent clinical trials. Treatment decisions increasingly incorporate patient and physician preferences regarding multiple aspects of quality of life, practicality, and cost‐effectiveness. This approach relies on alignment of patient and physician perceptions of symptoms and treatment of ITP, and clarity of expectations.

The international ITP World Impact Survey (I‐WISh) study was completed by patients with ITP and physicians experienced in treating them. The goals were to discern how ITP and associated treatments affect patients' lives, and to evaluate how aligned patient and physician perceptions are regarding symptoms, HRQoL and disease management. This primary I‐WISh report explores: (a) the diagnostic pathway for patients with ITP, and (b) patient and physician perceptions of symptoms and disease management. The HRQoL results are reported in the companion manuscript.^40^

## 2 METHODS

### 2.1 Study design

A comprehensive summary of study design and methods is provided in supplemental materials Appendix S1. So, I‐WISh was an exploratory, cross‐sectional survey of patients with ITP and physicians who treat ITP from Canada, China, Colombia, Egypt, France, Germany, India, Italy, Japan, Spain, Turkey, UK, and USA. Patients ≥18 years old diagnosed with ITP were recruited. Hospitalized patients were excluded. Patient survey invitations were sent via email to patient association groups (PAG) and physicians, who were responsible for further dissemination to patients either during routine consultations, targeted email, word of mouth, and/or personal contact in PAG meetings. Physicians were asked to invite patients on a consecutive basis but were not required to report the proportion who chose to participate.

Physicians (hematologists/hemato‐oncologists) with a minimum, active caseload of three patients with ITP and responsible for treatment decisions when completing the survey, were recruited via local fieldwork agencies (independent contractors) with specialized disease area knowledge.

Parallel patient and physician surveys were developed by a steering group comprising expert ITP clinicians and PAG leads. The patient questionnaire comprised six sections and requested information on demographics and diagnosis (seven questions), ITP symptoms (four questions), HRQoL and emotional impact (12 questions), impact on work, finances, and support (15 questions), treatment received (17 questions), and patient‐physician relationship (seven questions). The physician survey comprised six sections that collected information on demographics (two questions), ITP diagnosis and patient caseload (seven questions), ITP symptoms (five questions), impact of ITP on aspects of patients' physical, emotional, HRQoL, and social health (11 questions), treatment patterns (13 questions), and patient‐physician relationship (four questions).

Patients also completed the 10‐question ITP Life Quality Index^31^ on: working life or school, time taken off work or education, ability to concentrate, social life, sex life, energy levels, ability to undertake daily tasks, ability to provide support, hobbies, and capacity to exercise. Response options were either based on a 4‐point scale eg ”never”, ”sometimes”, ”more than half the time”, and ”all the time”, or on a 7‐point Likert scale, where 1 = ”never” and 7 = ”a great deal”.

The 30‐minute surveys (supplemental data Appendix S1) were provided to participants either online or by hard copy. Fully de‐identified respondent information was collated and aggregated by local fieldwork partners; thus, surveys were unlinked and anonymized. Survey materials and protocol were reviewed and approved by the Western Institutional Review Board. Patients and physicians were given an overview of the study and ethical approval details; those who wished to participate had to provide consent via a tick/check box before initiating.

### 2.2 Statistical analyses

Patient and physician surveys were analyzed separately; there was no linkage between patients and physicians. There were no pre‐specified hypotheses associated with these exploratory surveys, and as such, data were summarized narratively using descriptive statistics. For numeric variables, the respondent base, mean, and range (minimum and maximum values) were reported. For categorical variables, the total number and percentage of responses are shown. Participants with missing data were removed from all data summaries associated with that variable, but were still eligible for inclusion in other data summaries. Statistical significance analyses were not conducted.

Analyses were performed by subgroups according to sex (males vs females), age (18‐34, 35‐49, 50‐64, ≥65 years), and symptom burden (low, moderate, high, very high). Overall symptom burden score was calculated at diagnosis and survey completion separately by summarizing individual patient‐reported symptom severity scores, which were then divided into quartiles where Q1 = lowest symptom burden, and Q4 = highest symptom burden (supplemental methods Appendix S1).

## 3 RESULTS

In total, 472 physicians and 1507 patients from 13 countries completed the survey between December 2017 and August 2018. Patients were recruited by experienced ITP physicians (43%) or patient association groups (PAG; 57%), with variation by country (Table S1). Reliable estimates of how many people were contacted to participate in the survey could not be obtained.

Sixty‐five percent of patients were female, with mean age 47 years and median duration of ITP 5 years (Table 1); baseline characteristics were similar across sexes and age groups evaluated (data not shown). Most physicians were university/teaching hospital‐based (56%), qualified during the 20‐year period 1994‐2014 (72%), and self‐described as hematologists (66%), not hematologist‐oncologist. Physicians reported mean (SD) ITP patient caseload of 34 (50) and 18 (36) newly‐diagnosed patients in the past year (Table 1). Sixty‐four percent of patients rated their current health state as high (5‐7 on a 7‐point Likert scale where 1= very poor health, 7= excellent health), nonetheless 48% reported that they had high or very high symptom burden at diagnosis (defined in supplemental methods Appendix S1).

**TABLE 1:** Participant demographics and baseline characteristics

**Variable**	**Patients**
	**N = 1507**
**Mean** **(SD) age** ^**a**^, **years**	46.9 (16.22)
**Mean (SD) age of retired ITP patients, years (n = 278)**	68.2 (7.66)
Female, n (%)	975 (65)
**Median (IQR) length of time with ITP, years**	5 (2–12)
**Patient self‐reported current health state (n = 1503)** **(7‐point Likert scale; 7 is excellent health), n (%)**	
1–3 (low)	225 (15)
4	321 (21)
5–7 (high)	957 (64)
**Route of patient recruitment, n (%)**	
Patient association group‐invited	855 (57)
Physician‐invited	652 (43)
**Splenectomized, n (%) (n = 1325)**	263 (20)
**Symptom burden at diagnosis, n (%) (n = 1234)**	
Low	374 (30)
Moderate	270 (22)
High	282 (23)
Very high	308 (25)
**Current employment status, n (%)**	
Working full‐time	661 (44)
Working part‐time	235 (16)
Retired	278 (18)
Homemaker	102 (7)
Student	68 (5)
Not working, not seeking employment	66 (4)
On long‐term sick leave or on disability	48 (3)
Not working, seeking employment	37 (2)
Other (no details)	12 (1)
	**Physicians**
	**N = 472**
**Specialty, n (%)**	
Hematology	313 (66)
Hematology‐oncology	159 (34)
**Current ITP caseload**	
Low (0–11 patients)	154 (33)
Moderate (12–30 patients)	146 (31)
High (31+ patients)	172 (36)
**Patient caseload (ITP and non‐ITP), mean (SD)**	454 (643)
**ITP patient caseload, mean (SD)**	
ITP patients at time of survey completion	34 (50)
ITP patients in the 12 months prior to survey completion	43 (70)
Newly‐diagnosed ITP patients in the 12 months prior to survey completion	18 (36)
**Clinical setting, n (%)**	
University/teaching hospital	265 (56)
Regional/community hospital	121 (26)
Private hospital	39 (8)
Specialist cancer center	32 (7)
Other	9 (2)
Office‐based	6 (1)
**Year qualified, n (%)**	
Before 1981	11 (2)
1981–1993	95 (20)
1994–2003	172 (36)
2004–2014	171 (36)
After 2014	23 (5)

a N = 1506.

Abbreviation: ITP, immune thrombocytopenia; IQR, interquartile range.

### 3.1 Time from initial presentation to diagnosis of ITP

Patient‐reported median time from initial presentation to a healthcare professional (HCP) and ITP diagnosis (n = 1423) was 0.5 months (IQR 0.1‐1.0 month), with little difference between women, men, older and younger adults (supplemental data Appendix S1). Time from initial presentation to ITP diagnosis was >6 months for only 8% (n = 109/1423) of patients, and >12 months for 4% (n = 56/1423). In 455 (30%) patients who experienced a diagnosis delay ≥1 month, the most frequent signs and symptoms (>20%) were fatigue (50%), purpura (37%), anxiety around unstable platelet count (34%), and petechiae (29%). Those diagnosed with ITP within 1 month had more overt bleeding (purpura 63%, gum bleeding 36%, and epistaxis 33%).

Patient initial presentation was frequently to a general practitioner (52%), whereas diagnosis was almost always made by a hematologist (86%) (Figure S1). Two‐thirds (66%) of patients were referred to another HCP after initial presentation; the median number of physicians seen before ITP diagnosis was 2.0 (range 1‐10). The mean (SD) number of symptoms reported at diagnosis was five (3.1) and this was similar whether the patient had seen one (4.7) or three physicians (5.2).

### 3.2 Diagnosis

The most common diagnostic tests that physicians recalled using in patients suspected of ITP, and depending on symptom burden (assessed as asymptomatic, low, moderate or high symptom burden at the investigators' discretion), were complete blood count (79%‐83%), physical examination (75%‐82%) and peripheral blood smear (71%‐74%). Physicians reported they would perform bone marrow biopsy/aspirate most frequently in patients they judged to have high symptom burden (73%), and less so in patients with moderate (52%) or low symptom burden (26%).

Physicians believed delays in diagnosis of ITP resulted from excluding other causes of thrombocytopenia (68%; n = 319/472), time waiting to see a specialist (58%; n = 274/472), diagnostic examination (55%; n = 261/472), and misdiagnosis as another condition (53%; n = 249/472). Seventy‐one percent of physicians believed that up to one‐quarter of patients were misdiagnosed (only 7% believed there were no misdiagnoses); this was consistent across countries except for Germany where 43% of physicians believed 51%‐75% of patients were misdiagnosed. Misdiagnoses were most frequently thought to be drug‐induced thrombocytopenia (67%), aplastic anemia or myelodysplastic syndromes (49%), liver disease (46%), hypersplenism (38%), or leukemia (29%).

Twenty‐two percent (n = 329) of patients reported delayed diagnosis and felt this was caused by waiting for additional tests (48%; n = 159/329) or referral to a specialist (not specified) (36%; n = 119/329); or both (18%; n = 58/329). Among patients who experienced diagnosis delay, 73% (n = 241/329) were anxious (scored ≥5 on a Likert scale 1‐7; 7 = extremely anxious), of whom 36% felt extremely anxious (scored 7; n = 117/329).

Following diagnosis, sources of patient support were family and friends (59%; n = 890/1507), physicians (52%; n = 789/1507), both consistent across countries and, where available, a PAG (27%; n = 410/1507). Patients with very high symptom burden sought support from a PAG more frequently (35% vs 23% with low symptom burden). Support at diagnosis was similar by sex and age subgroups (supplemental data Appendix S1).

Overall, 63% (n = 945/1507) of patients wished they had received more support at diagnosis, most often from their physician (60%; n = 569/945) or a PAG (50%; n = 468/945). The exceptions were in China, where 96% of patients wanted more support during diagnosis, mostly from their physician (80%), and Egypt and Japan where only 13% and 14% of patients wanted more support. In seven countries, 5% of patients or less received support from a PAG at diagnosis yet this was what 49% of patients from these countries wanted. Patients with very high/high symptom burden wanted more support at diagnosis (75% and 76% vs 58% with low symptom burden).

### 3.3 ITP symptoms and severity

**FIGURE 1:**
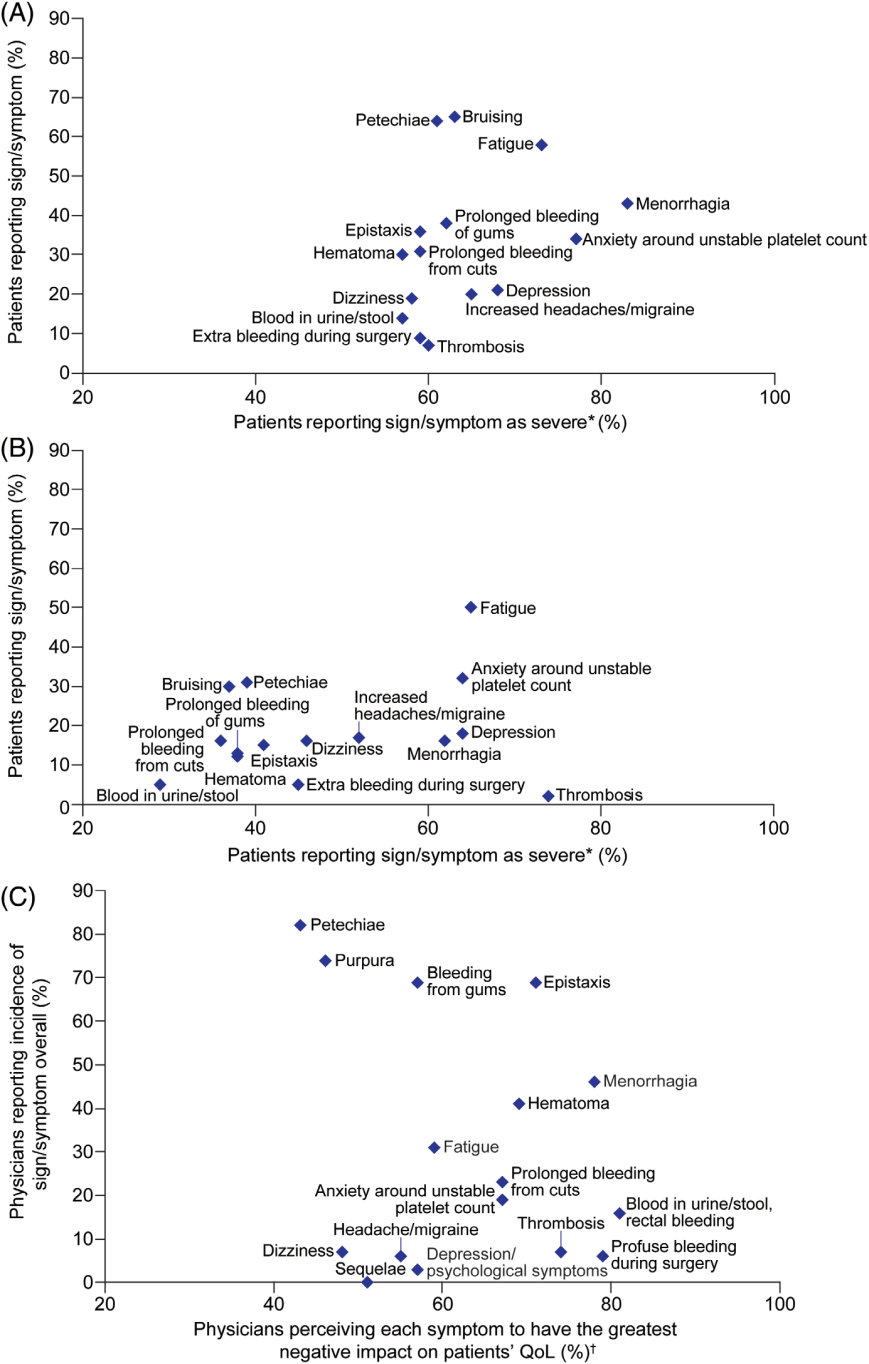
(A) Patient perception at diagnosis, and (B) time of survey completion and (C) physician perception overall of occurrence and severity of ITP signs and symptoms. *Patients reported symptom severity on a 7‐point Likert scale where 1 = not severe at all and 7 = worst imaginable. The data presented are for”severe” based on scores ≥5. Severity of each symptom was only rated if patients stated that they had experienced it at the time of ITP diagnosis and survey completion. ^†^Physicians (n = 472) reported the top five symptoms they most frequently heard from patients and reported symptom effect on patients' QoL on a 7‐point Likert scale where 1 = not at all and 7 = a great deal. Data presented are for scores ≥5. ITP, immune thrombocytopenia; QoL, quality of life [Color figure can be viewed at wileyonlinelibrary.com]

Petechiae (64%, diagnosis; 31%, survey completion) and bruising of unknown origin (65%, diagnosis; 30%, survey completion) were among the most frequent patient‐reported signs and symptoms at diagnosis and, though substantially reduced, were prevalent at survey completion (Figure 1A,B). In contrast, anxiety around maintaining a stable platelet count (34%, diagnosis; 32%, survey completion) and fatigue (discussed below) were also frequent. These four were the top signs and symptoms that patients most wanted resolved. Very few patients reported no symptoms at diagnosis (6%) or survey completion (13%).

Anxiety around maintaining stable platelet counts did not change over time, and patients who experienced it rated it as one of their most severe symptoms (scored ≥5 on a Likert scale 1‐7; 7 = the worst imaginable), at diagnosis (77% with n = 400/517) and survey completion (64% with n = 303/474) (Figure 1A,B). Thrombosis had occurred in 103 patients at diagnosis and 35 at survey completion, patients who experienced thrombosis considered it as one of their most severe complications (60%, n = 62/103 and 74%, n = 26/35; Figure 1B). Heavy menstrual bleeding (HMB) was experienced by 439 women at ITP diagnosis and 161 at survey completion, of whom 83% (n = 364/439) and 62% (n = 100/161) rated it as one of their most severe symptoms.

The most common signs and symptoms physicians reported hearing about from their patients at any time (overall) and at diagnosis were similar to those reported by patients, most frequently petechiae (82% overall; 83% at diagnosis), purpura (74%; 73%), bleeding gums (69%; 70%), and epistaxis (69%; 70%) (Figure 1C). Physicians also believed several signs and symptoms have a major negative impact on patient HRQoL (scored ≥5 on a Likert scale 1‐7; 7 = a great deal; Figure 1C): hematuria, melena or rectal bleeding (81%), profuse bleeding during surgery (79%), and menorrhagia (78%).

Fatigue was one of the most frequent patient‐reported signs and symptoms at diagnosis (58%) and especially at survey completion (50%) (Figure 1A,B). More women than men reported fatigue at diagnosis (61% vs 52%) and at survey completion (54% vs 42%). Similar proportions of younger patients (18‐34 years) and patients aged >65 years reported fatigue at diagnosis (56% vs 51% respectively) and time of survey completion (46% vs 48% respectively), suggesting fatigue was not a phenomenon of aging. At diagnosis and survey completion, respectively, 73% (n = 637/870) and 65% (n = 487/752) of patients reporting fatigue considered it severe (scored ≥5 on a Likert scale 1‐7; 7 = the worst imaginable); furthermore, fatigue remained consistently frequent and severe over time (Figure 1B). Of the signs and symptoms patients were experiencing when completing the survey, 46% wanted fatigue to be resolved. Whilst bleeding and bruising decreased with management of ITP, fatigue did not undergo a similar reduction; no pattern was observed between platelet count and fatigue at survey completion.

Physicians reported hearing about fatigue from their patients less frequently (31% overall; 30% diagnosis) than patients reported experiencing fatigue (58% diagnosis; 50% survey completion). Physicians with higher ITP caseload more frequently reported fatigue (37% diagnosis, 40% overall) than physicians with lower ITP caseload (18% and 23%). Fifty‐nine percent of physicians believed that having fatigue would greatly impact patient HRQoL (Figure 1C).

**FIGURE 2:**
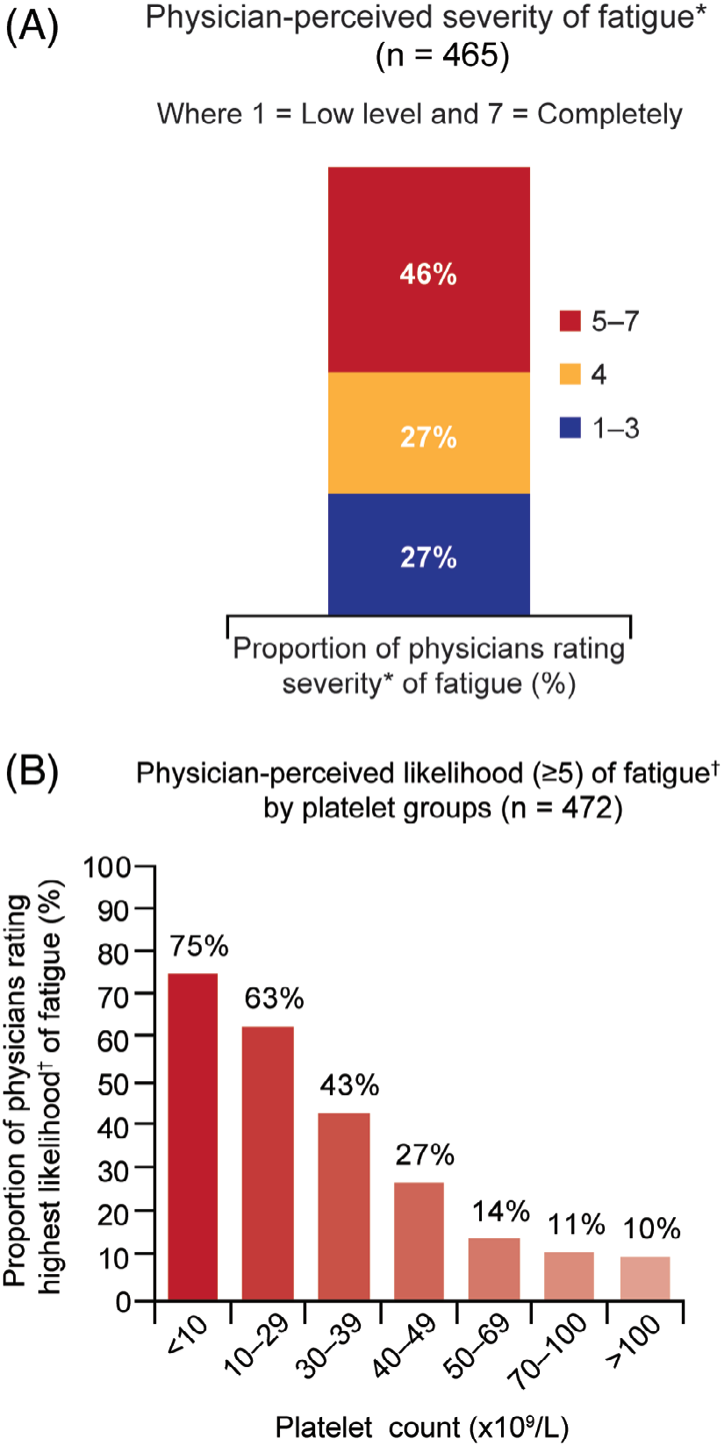
(A) Physician perception of their patients' severity of fatigue, and (B) likelihood of fatigue by platelet group. *Physicians reported perceived severity of fatigue on a 7‐point Likert scale where 1 = low level and 7 = completely.^†^Physicians reported likelihood of fatigue on a 7‐point Likert scale where 1 = not at all and 7 = very likely [Color figure can be viewed at wileyonlinelibrary.com]

Physicians (n = 465) reported that 38% of their patients felt fatigued and considered that 46% of these experienced significant fatigue (score ≥ 5 on a Likert scale 1‐7; 7 = completely fatigued). Most physicians believed that platelet counts <10 × 10^9^/L were associated with increased fatigue severity (75%; n = 355/472) compared with platelet counts >100 × 10^9^/L (10%; n = 47/472) (Figure 2A,B).

### 3.4 Treatment and management

Physicians indicated that during the first 6 months following diagnosis, ”watch and wait” was the preferred option for 39% of patients, rather than pharmacological intervention; it was physicians' preferred option for 34% of previously‐treated patients with chronic ITP. Factors (>50%) influencing ”watch and wait" instead of recommending drug treatment were higher platelet levels (89%; n = 412), patient being asymptomatic (75%; n = 348), and absence of severe bleeding symptoms (59%; n = 273). Over half the patients (54%; n = 806/1501) reported they had been on ”watch‐and‐wait” management at some time, with no clinically meaningful differences between the sexes or age subgroups (data not shown).

#### 3.4.1 Splenectomy

Physicians were asked what proportion of patients, in their experience, would undergo splenectomy. Physicians estimated 10% of newly‐diagnosed ITP (prior to relapse) would undergo a splenectomy whereas for persistent, chronic or recurrent ITP the estimate was 32%. One‐fifth of patients reported that they had undergone a splenectomy (20%; n = 263/1325); 13% of younger patients (18‐34 years old) and 28% of older patients (≥65 years old) had been splenectomized. The highest proportions of patients having had a splenectomy were in the US (34%) and Canada (33%) and the lowest in China (6%) and India (9%).

#### 3.4.2 Pharmacological treatments

**FIGURE 3:**
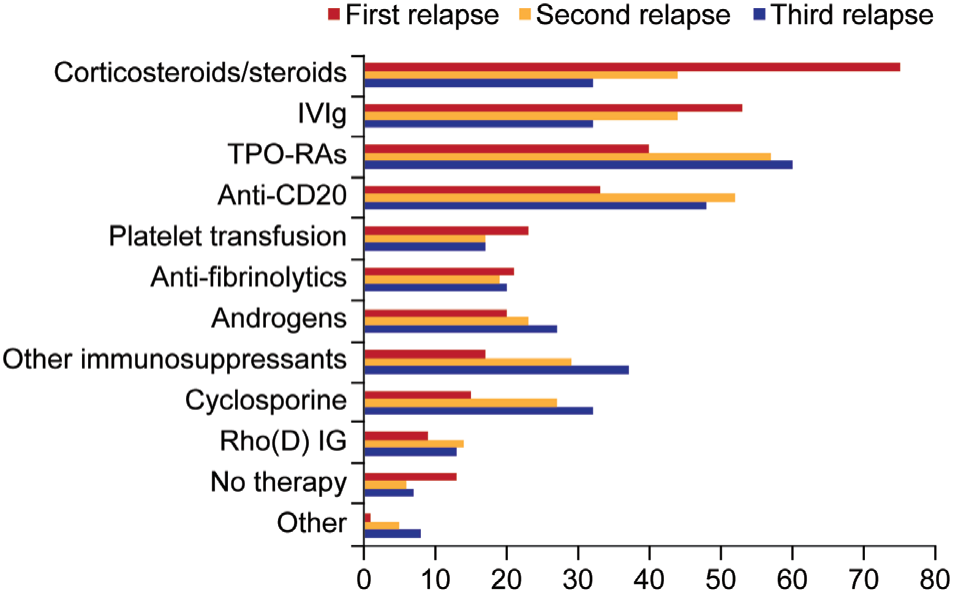
Treatment prescribed by physicians following relapse of ITP. ITP, immune thrombocytopenia; IVIg, intravenous immunoglobulin; TPO‐RA, thrombopoietin receptor agonist [Color figure can be viewed at wileyonlinelibrary.com]

Just under two‐thirds (64%; n = 968/1501) of patients felt their symptoms and HRQoL were considered when treatment decisions were made. The three most commonly prescribed treatments for newly diagnosed patients were corticosteroids or steroids, such as prednisolone, methylprednisolone or dexamethasone (89%; n = 418/472), IVIg (65%; n = 305/472), and anti‐fibrinolytics (35%; n = 163/472); however, use of IVIg and anti‐fibrinolytics varied considerably between countries. For persistent, chronic or recurrent ITP, the three most common treatments physicians would prescribe were TPO‐RAs (80%; n = 377/472), anti‐CD20 (76%; n = 357/472), and ”other immunosuppressants” (55%; n = 260/472). At first relapse, physicians would most commonly prescribe corticosteroids or steroids (75%; n = 355/472). At second and third relapse, TPO‐RAs (57%; n = 269/472 and 60%; n = 281/472), and anti‐CD20 (52%; n = 245/472 and 48%; n = 228/472) were selected (Figure 3).

The reasons for physicians changing therapy were lack of efficacy (87%; n = 411/472), disease worsening (74%; n = 350/472), treatment‐related side effects (76%; n = 358/472), blood count change (45%), cost (35%), patient preference (34%), and symptom change (33%); no physician selected ”other” reason. In choosing treatments, physicians and patients prioritized the ability to offer sustained remission (82% and 90%), reduction in bleeding risk (86% and 87%), fewer side effects (76% and 86%), and avoiding immunosuppression (61% and 72%).

Platelet count control was important for patients, with poor control having a negative effect on emotional well‐being (acknowledged by physicians). Only 23% (n = 345/1507) of patients reported stable platelet counts over the previous 2‐6 months. Platelet counts were checked every 1.2 (3.2) and 2.1 (4.3) months for newly‐diagnosed and persistent, chronic, or recurrent ITP patients.

Physicians rated the top three treatment goals for their patients as reduction of spontaneous bleeds (72%; n = 340/372), improving quality of life (64%; n = 302/472) and healthy blood counts (51%; n = 241/472). The patient survey also identified healthy blood counts within the top three treatment goals (64%, n = 965/1507). Preventing episodes of worsening of ITP (44%, n = 663/1507) and increasing energy levels (41%, n = 618/1507) were the other two top three goals identified by patients. Seventy‐one percent (n = 1070/1507) of patients ”somewhat” or ”strongly” agreed that their current treatment was helping them reach their treatment goals and 74% (n = 1115/1507) felt that their doctor was aware of their treatment goals.

## 4 DISCUSSION

I‐WISh extensively explored the degree of symptoms associated with ITP from the patients' perspective for the first time and identified those that patients most wanted resolved. Also for the first time, patient and physician perceptions were evaluated together and were generally aligned, with similar signs and symptoms and use of treatments being reported, despite the patients not being those of the participating physicians.

More than half of patients reported their current health as ”high”. This may reflect a perceived normalization of their disease burden over time, for example, getting acclimatized to certain conditions, although they had not changed, and/or developing ”work arounds”. The true burden of disease became apparent with almost half (48%) of respondents indicating a high or very‐high symptom burden, even though some of these respondents had nonetheless also indicated good or very good health. Similarly, despite patient anxiety about platelet counts, and only 23% saying platelet counts had been stable for 2‐6 months, counts were not checked on average more often than every 1‐2 months, potentially reflecting a disconnect in some patients between physicians' and patients' perceptions of the clinical stability of their ITP, resulting in patient anxiety about their disease.

Time to diagnosis was less than a month in three‐quarters of patients, even though most cases required referral to a hematologist and ITP diagnosis is made by exclusion of other causes of thrombocytopenia. Diagnosis was delayed in patients who reported more diverse symptoms such as fatigue when compared with patients who primarily had overt bleeding. Cases presenting with more severe systemic symptoms would not be immediately recognized as ITP; a previous study has highlighted an almost 20% incidence of misdiagnosis among experienced hematologists.^15^

Petechiae and bruising were two of the most frequent patient‐reported symptoms at diagnosis but were reduced by half at survey completion, a median of 5 years later. However, two other frequent patient‐reported symptoms at diagnosis, fatigue and anxiety about stable platelet counts, did not substantially decrease over time. It is not clear whether this was caused by lack of treatment, failure to respond to it, or patient anxiety independent of the state of the ITP. The fall in bleeding symptoms in ~50% of patients (and even greater reductions in HMB) suggests that persistence of fatigue and platelet count anxiety were likely independent of the platelet count, although it might not have been the same patients reporting fatigue and anxiety at diagnosis and survey completion. The anxiety experienced around maintaining stable blood counts requires further research to establish if it is intrinsic or arises from external sources such as a physician, family members, or the internet. There was likely an inadvertent bias in that patients choosing to participate may have done so because they continued to experience these issues. The proportion of patients experiencing each ITP symptom and its reported severity did not differ greatly among the subgroups analyzed, suggesting that the burden of ITP affects all patients to a similar extent whether men or women, younger or older adults. This was unexpected given the likelihood that fatigue would be more prevalent in older age; this may also speak to the need to reset expectations of "normal” among patients with ITP.

For patients within 6 months of diagnosis and with mild symptoms, physicians indicated a preference for ”watch‐and‐wait”. Pharmacological treatments most commonly prescribed either at diagnosis or first relapse were corticosteroids and IVIg; both were used for first relapse but use decreased after second relapse. For patients with persistent/chronic disease or after subsequent relapse, TPO‐RAs and anti‐CD20 were most commonly prescribed, with splenectomy relatively infrequently selected; however, it had been performed in one‐fifth of participating patients, with wide national variability. In those nations perceived to have more resources, for example, the United States, splenectomy rates were higher than in India and China, which was surprising. Again, this may have reflected the bias of patients who entered the study.

### 4.1 Fatigue is often persistent and severe

The findings of I‐WISh expand on a smaller previous survey of Japanese physicians and patients with ITP^7^ that also reported high fatigue occurrence.^6^, ^7^, ^8^ In I‐WISh, fatigue was one of the top three symptoms (along with bleeding and unstable platelet counts) that patients most wanted resolved. Physicians considered fatigue as important, but they reported it substantially less often than did patients; physicians noted its role in impairment of quality of life and prioritized increasing energy levels when fatigue was present. Physicians believed fatigue would decrease as platelet count increased, and thus that fatigue was intrinsically related to disease activity. However, fatigue persisted, despite amelioration of other components of ITP between diagnosis and survey completion, suggesting that increasing platelet count and reducing bleeding is not sufficient to abrogate fatigue. Indeed, a recent study of fatigue in children and adolescents with ITP showed that fatigue scores did not correlate with bleeding symptoms, platelet count, or platelet response to treatment.^16^

Underestimation by physicians of fatigue frequency in patients with ITP, although reported more often by physicians with longer clinical experience, has many potential causes. On the one hand, it could reflect an ”insufficient” assessment in patients as the visit time may be focused on issues such as bleeding or other medical concerns. Alternatively, patients may not always vocalize their experiences of fatigue, which could also be compounded by the large proportion of patients describing good health. These results highlight the importance of the physician fully inquiring into the degree of fatigue, including raised anxiety levels and other causes, and also patients being more forthcoming about their daily life experiences and not accepting sub‐optimal ”normal”.

The best approach to treating fatigue remains unresolved but, if present, attempts at amelioration should certainly be made. Among many options, raising platelet count (ideally without using steroids), antidepressants and/or anti‐inflammatories could be considered. Clinical trials in this area are needed.

### 4.2 Treatment landscape

Corticosteroid therapy, with or without IVIg, is standard first‐line ITP treatment. Accordingly, I‐WISh found that 90% of physicians prescribe corticosteroids for newly‐diagnosed patients and 75% use them for first‐relapse patients; 79% of patients reported that they had received corticosteroids. It is well known that prolonged corticosteroid exposure has many detrimental effects,^17^, ^18^ which is reflected in the American Society of Hematology (ASH) guidelines and International Consensus document recommendations to restrict steroid use to the short term and not to prescribe repeated courses.^2^, ^9^ These ITP‐experienced physicians mostly initiated TPO‐RAs and anti‐CD20 for patients with persistent or chronic disease needing treatment and for those experiencing a second or third relapse (third or fourth episode of ITP). The use of corticosteroids (and IVIg) was substantially reduced but not halted.

Numerous second‐line treatment options are available, but there is no clear consensus on the optimal order of use. This results in marked variability in ITP management after first relapse, as reflected here by retained use of platelet transfusion, androgens, and anti‐fibrinolytics, as well as the increased use of other immunosuppressants, cyclosporine, TPO‐RAs, and anti‐CD20. Safety and efficacy of TPO‐RAs and anti‐CD20 in ITP have been demonstrated in many single‐arm clinical trials,^19^, ^20^, ^21^, ^22^, ^23^, ^24^, ^25^, ^26^, ^27^, ^28^, ^29^, ^30^, ^31^ with some studies highlighting the potential for treatment‐free remission following TPO‐RA discontinuation;^32^, ^33^, ^34^, ^35^ and similar effects seen with anti‐CD20.^36^, ^37^, ^38^, ^39^ Although use of these agents in “real‐world” practice changes frequently, I‐WISh indicates that TPO‐RAs and anti‐CD20 have become widely used in patients with ongoing disease.

Despite I‐WISh being the largest survey of its kind, including physicians and not just patients, the limitations include the inability to estimate response rate, and potential biases introduced inadvertently because some patients were more motivated to participate than others, for example, those with high levels of anxiety or more severe disease (noting that only 23% of participating patients indicated stable platelet counts) vs patients with very low levels of fatigue and/or fewer concerns about their ITP. Additionally, the questions posed had fixed, multiple choice, or Likert scale responses (not open‐ended questions with free‐text answers) and there was no opportunity to explore respondent answers further; these factors limit the level of interpretation. For example, although patients were asked whether they experienced fatigue and, if so, how severe it was, no additional questions were asked to gather extra insight regarding what their fatigue specifically related to. As with any questionnaire, participants could have misinterpreted questions.

There was no patient identifying linkage between survey administration and survey completion, thus we were unable to link responses and clinical data. Therefore, disease severity was unknown in patients who completed the questionnaire compared with those who did not. Furthermore, because no clinical data were collected neither patient nor physician responses can be verified; this is particularly pertinent considering patients were required to remember their condition at diagnosis, which was a median of 5 years before completing the survey. Nonetheless, group responses were very valuable for understanding different aspects. Lastly, there was no concurrent control group. Many of these limitations will be addressed in I‐WISh 2.0, scheduled to begin before the end of 2020.

All participating physicians had experience in ITP, and likely more awareness of both the burden of ITP on patients' lives and the benefits and limitations of available treatment options than most hematologists and hematologists‐oncologists in general practice. As a consequence, their responses may have been more knowledgeable than those obtained from a group of less‐experienced, more ”typical” hematologists and hematologists‐oncologists. Additionally, geographical variation and availability of treatments and other resources, for example, specialists, likely influenced the results, particularly regarding delays in diagnosis and treatment selection.

Despite its limitations, the I‐WISh survey provides a strong signal of the extent of symptoms associated with ITP from the patients' and physicians' perspectives, including both frequency and severity. Strengths were the large sample size, broad scope of topics pertinent to the lives of patients with ITP and acquisition of both physician and patient perspectives, illustrating where misperceptions occurred and ideally would be avoided in the future. Thus, I‐WISh identified those issues that patients most want resolved and provided insight into real‐world treatment patterns and patient experience with them. I‐WISh has also extensively explored the functional and psychological/emotional impact of ITP on HRQoL, the impact on productivity, treatment satisfaction and the patient‐physician relationship, these results are reported elsewhere (Cooper et al., manuscript in preparation).

## 5 CONCLUSIONS

ITP is a serious condition which, even if infrequently fatal (thanks at least in part to an array of effective treatments), has a substantial adverse impact on the quality of many patients' daily lives. Fatigue was a frequently reported, persistent and severe symptom associated with ITP, which at best did not appear to consistently respond to platelet‐increasing. Although participating physicians were experienced in ITP management and believed that fatigue greatly affects patients, they reported fatigue less frequently than did patients, likely reflecting multiple etiologies. Patients indicated the need for considerable support from multiple sources, especially at diagnosis, not only from physicians, family and friends, but also other patients highlighting the important role for PAGs. These findings should be integrated into the clinical management of each patient with ITP in an individual fashion according to each patient's needs. Further research, including I‐WISh 2.0, is needed to assess and hopefully verify these exploratory findings through an appropriately powered, hypothesis‐driven methodology linking the clinical to the quality of life and fatigue‐related issues.

## CONFLICT OF INTEREST

N. Cooper reports honoraria for speaking engagements and advisory boards from Amgen and Novartis. C. Kruse received honoraria for speaking engagements and consultancy fees paid to PDSA from Amgen, Novartis, and Rigel Pharmaceuticals. S. Watson reports advisory work for Novartis. M. Morgan, reports advisory work for Novartis, Sobi and UCB. D. Provan received research grants and honoraria from Novartis and Amgen and consultancy for UCB, MedImmune, and ONO Pharmaceutical. W. Ghanima received research grants from Bayer, BMS, and Novartis and honoraria for participation in advisory boards for Amgen and Novartis. D. M. Arnold received research grants from Novartis, Amgen, and Bristol‐Myers Squibb and worked as a consultant for Amgen, Novartis, Rigel, and Principia. Y. Tomiyama reports honoraria and membership of advisory committees for Novartis and honoraria from Chugai and Kyowa‐Kirin. C. Santoro reports participating in speakers' bureaus for Amgen, advisory boards for Grifols and Gilead, and speakers' bureaus and advisory boards for Shire/Takeda, Novo Nordisk, Bayer, Pfizer, CSL, Roche, Novartis and Sobi. M. Michel reports membership of advisory boards and speaker engagements for Amgen and Novartis. B. Lovrencic reports honoraria for consultancy fee paid to Aipit (Italian Association of Immune Thrombocytopenic Purpura) from UCB and Novartis. J.B. Bussel reports consulting or membership on an advisory board for Amgen, Novartis, Dova, Rigel, UCB, Argenx, Kezar, RallyBIo, and Momenta; honoraria from UptoDate, and participating in a speakers' bureau for Novartis, 3S (Shenyang) Bio, and Physician Education Resource. J. Haenig is a full‐time employee of Novartis Pharma AG. T. Bailey and G. Taylor‐Stokes are employees of Adelphi Real World, which has received consultancy fees from Novartis. A. Kruse, S. Laborde, and M. Hou have nothing to disclose.

## AUTHOR CONTRIBUTIONS

All authors contributed to the design of this study, raised awareness, recruited patients and interpreted the data; T.B. and G.T.S. coordinated data collection and analysis; and all authors critically reviewed the draft and approved the final version for publication.

## Supporting information


**Appendix** **S1**: Supporting information

## Data Availability

Novartis is committed to sharing with qualified external researchers access to patient‐level data and supporting clinical documents from eligible studies. These requests are reviewed and approved by an independent review panel on the basis of scientific merit. All data provided are anonymized to respect the privacy of patients who have participated in the trial, in line with applicable laws and regulations. This trial data availability is in accordance with the criteria and process described on www.clinicalstudydatarequest.com
