# Supplementary material for: Immune thrombocytopenia (ITP) World Impact Survey (iWISh): Patient and physician perceptions of diagnosis, signs and symptoms, and treatment
Source: Am J Hematol. 2020 Dec 19;96(2):188–98. doi: 10.1002/ajh.26045 (PMC7898610; doi:10.1002/ajh.26045)
Supplement: Supplementary file 1 — Appendix S1: Supporting information [file AJH-96-188-s001.docx]

###### **Immune thrombocytopenia (ITP) World Impact Survey (I-WISh): patient and physician perceptions of diagnosis, signs and symptoms, and treatment**

**Supplemental Data**

**I-WISH rationale, participants, study design and methods**

**Rationale**

The aim of the I-WISh survey was to collect information from physicians and patients to elicit understanding of the disease impact of ITP in terms of symptom, emotional and economic burden, impact on daily living and work productivity and patient/physician perceptions and disconnects on disease treatment and management.

We planned to explore the patient voice, including patients with ITP who did not regularly consult with a physician. We planned to include physicians with a breadth of experience in treating patients with ITP.

We did not plan to link clinical and patient responses but maximized recruitment to explore the patient voice and to identify what aspects of the diagnosis and treatment pathway were important to the patient. Identification of these key factors may help improve the treatment experience and consequently clinical outcomes.

**Participants**

Physicians – Physicians who actively treated and managed patients with ITP and had a minimum caseload of 3 ITP patients in the 12 months preceding the survey.

Patients – Patients aged ≥18 years, with a diagnosis of ITP willing and able to provide consent. Patients with advanced ITP disease who were hospitalized were excluded.

**Study design**

This was an exploratory, cross-sectional study that administered questionnaires to both physicians who treated patients with ITP and patients diagnosed with ITP.

The questionnaires are independent of each other and there are no matching questions

The main components of the physician questionnaire were:

- Physician demographics
- Patient caseload
- ITP diagnosis pathway
- Patient symptomology
- Impact of disease for the patient
- Treatment patterns
- Physician-patient relationship

The main components of the patient questionnaire were:

- Patient demographics and disease characteristics
- Impact of disease (symptom, emotional, financial)
- ITP diagnosis pathway (including associated burden)
- Quality of life
- Impact of ITP on daily living and productivity
- Treatment received and attitudes towards disease and treatment
- Physician-patient relationship

**Methods**

Data for both questionnaires were collected between December 2017 and August 2018) from physicians and patients in 13 countries (Canada, China, Colombia, Egypt, France, Germany, India, Italy, Japan, Spain, Turkey, UK, and US).

The physician and patient questionnaires were translated into an appropriate language by an accredited translation agency and were proof-read by a native speaker (within each local fieldwork partner) for each language. Review and reconciliation of translations took place until they were approved and certified by the native speaker.

No clinical data were collected from patient or physician records. Due to the anonymity of the survey, and to maximize the patient voice, no links were possible between patient and physician surveys.

Both surveys took approximately 30 minutes for respondents to complete.

Data collection was conducted by Adelphi Real World (ARW) through local fieldwork partners in each country.

Physician survey: Local fieldwork partners provided physicians with information about the online survey and instructions about completion. Providing a link and using fieldwork partners ensured that neither ARW nor Novartis were aware of the identity of participating physicians. Each survey was identified with a survey number to ensure anonymity. The survey was piloted by one physician in each country.

Patient survey: Physicians or patient advocacy groups (PAGs) approached patients, diagnosed with ITP, and invited them to complete the survey. Physicians were identified by ARW to facilitate the recruitment of ITP patients. Local fieldwork partners provided physicians with an overview of the survey. Physicians identified consecutive, eligible patients during routine consultations and provided them with information about the study and how to complete the questionnaire. Physicians were not asked to report on the number of patients who agreed to participate versus those who did not. Patients were also recruited through PAGs. ARW liaised with the advocacy group leads to ensure that survey links were only shared within communities where members were known to have ITP. The use of closed networks helps to maximise the legitimacy of respondents, while providing an opportunity to recruit a large number of patients with ITP. The patients from the PAGs included those who may not routinely consult with their physician and would, therefore, not have been otherwise represented in a matched physician-patient study design. Patients who were willing to participate were directed to a link if they were accessing the online survey. Each survey was identified with a survey number to ensure anonymity. The use of fieldwork partners, PAGs and providing a link to complete the surveys ensured that neither ARW nor Novartis were aware of the identity of any of the patient participants.

Participation was voluntary. The patient survey was completed and submitted online (n=1411), or with pen and paper where internet access was limited (Japan: 2 online surveys, 54 paper surveys; Spain: 6 online surveys, 42 paper surveys). Each individual paper survey was quality-checked by two members of the study team at Adelphi Real World, and double data entry was conducted as part of adding these paper surveys into the study database. All physician surveys were completed online. Patients recruited through PAGs had to self-confirm their diagnosis of ITP; patients recruited via a physician were patients with a confirmed diagnosis. To ensure the participants were those with a diagnosis of ITP, all participants had to confirm their diagnosis during a screening phase prior to completing the survey to establish their eligibility.

**Data management**

All responses were de-identified, collated and aggregated before analysis. Surveys could only be completed once. Data were transferred to a single electronic database. Analyses were conducted using Stata Statistical Software Version 15.0 or later (StatCorp, 2015. Stata statistical software: Release 16 College Station, Tx, StataCorp LP).

Participants with missing data were removed from all data summaries associated with that variable but remained eligible for inclusion in other data summaries.

**Data analysis**

As links to questionnaires were sent by third parties, there is no method of recording the number of questionnaires provided, not started or partially completed and not submitted. Details on response rates are not provided.

As there were no pre-specified hypotheses in this exploratory study, no statistical analyses were undertaken. Data were summarized narratively using descriptive statistics. For numeric variables, the respondent base, mean, and range (minimum and maximum values) or median and inter-quartile range were reported. For categorical variables, the total number and percentage of responses were reported.

**Supplementary Methods**

**Classification of symptom burden groups**

The symptom burden groups were defined as:

At diagnosis:

- Low burden: A symptom burden score of 0–13
- Moderate burden: A symptom burden score of >13–≤21
- High burden: A symptom burden score of >21–≤34
- Very high burden: A symptom burden score of >34

At point of survey completion:

- Low burden: A symptom burden score of 0–6
- Moderate burden: A symptom burden score of >6–≤11
- High burden: A symptom burden score of >11–≤19
- Very high burden: A symptom burden score of >19

To demonstrate the accuracy of symptom burden allocation by symptom severity scores, the average number of symptoms reported by patients in each burden group was:

At diagnosis

- Low burden: 2.1 symptoms
- Moderate burden: 3.9 symptoms
- High burden: 5.6 symptoms
- Very high burden: 9.1 symptoms

At point of survey completion:

- Low burden: 1.3 symptoms
- Moderate burden: 2.4 symptoms
- High burden: 3.5 symptoms
- Very high burden: 6.6 symptoms

**Results**

**Time to diagnosis in women, men, older and younger adults**

Women experienced a median (IQR) interval of 0.5 months (0.1–1.2) between initial presentation and diagnosis, while men experienced a median of 0.2 months (0.1–1.0); older adults (≥65 years old) reported a median of 0.5 months (0.1–1.4), whereas younger adults (18–34 years old) reported 0.2 months (0.1–1.0).

**Support at diagnosis in women, men, older and younger adults**

Fewer women than men reported being supported by a physician (49% vs 58% respectively) whereas more women reported support by a PAG (30% vs 22% respectively). Younger patients (18–34 years) more frequently reported support by a physician compared with patients aged >65 years old (56% vs 48% respectively). Similarly, more younger patients wanted greater support at diagnosis than older patients age (68% vs 55% respectively).

**Supplemental Table S1. Summary of survey sample**

| **Country** | **Physician sample,  n** | **Patient sample,  n** | **PAG vs physician recruitment** |
| --- | --- | --- | --- |
| Canada | 30 | 61 | 61/0 |
| China | 102 | 286 | 170/116 |
| Colombia | 25 | 51 | 0/51 |
| Egypt | 20 | 16 | 16/0 |
| France | 34 | 87 | 18/69 |
| Germany | 30 | 82 | 0/82 |
| India | 21 | 65 | 1/64 |
| Italy | 30 | 74 | 0/74 |
| Japan | 36 | 56 | 0/56 |
| Spain | 31 | 48 | 0/48 |
| Turkey | 21 | 60 | 0/60 |
| UK | 31 | 120 | 120/0 |
| US | 61 | 501 | 469/32 |
| **Total** | **472** | **1507** | **855/652** |

PAG, patient advocacy group.

**Supplemental Figure S1. The diagnostic pathway for patients with ITP**

**
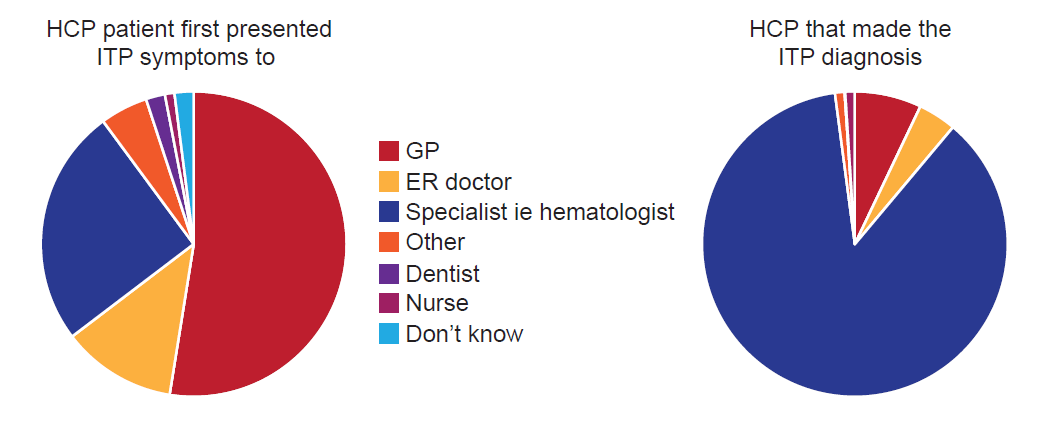
**

ER, emergency room; GP, general practitioner; HCP, healthcare professional; ITP, immune thrombocytopenia.
